# Food Safety Knowledge, Attitudes and Practices Among University Students in Rajasthan, India: A Cross‐Sectional Study

**DOI:** 10.1002/puh2.70364

**Published:** 2026-09-15

**Authors:** Naresh Kumar Sharma, Naveen Kumar, Ravinder Kaushik, Vijay Pal Singh, Sanjeeb Kumar Mishra

**Affiliations:** ^1^ Amity Institute of Biotechnology Amity University Rajasthan Jaipur Rajasthan India; ^2^ School of Agricultural Sciences K.R. Mangalam University Gurgaon Haryana India; ^3^ School of Health Sciences and Technology University of Petroleum and Energy Studies Dehradun Uttarakhand India; ^4^ CSIR—Institute of Genomics and Integrative Biology New Delhi India; ^5^ Maharajgunj Medical Campus Institute of Medicine Tribhuvan University Kathmandu Nepal

**Keywords:** foodborne disease, food safety behaviour, India, Knowledge–Attitude–Practice, university students

## Abstract

**Background:**

Foodborne diseases remain a major public health concern worldwide and are frequently associated with unsafe food‐handling practices and inadequate hygiene. University students often prepare or consume food independently and may lack sufficient awareness of hygienic food‐handling practices.

**Objectives:**

To assess food safety knowledge, attitudes and practices among university students in Rajasthan, India and to identify factors associated with hygienic food‐handling behaviour.

**Methods:**

A cross‐sectional study was conducted among 412 university students in Rajasthan, India, using a non‐probability convenience sampling approach through an online questionnaire. Data were collected using a structured, self‐administered questionnaire comprising items on sociodemographic characteristics, food safety knowledge, attitudes and practices. Knowledge was assessed using 12 statements with true, false and do not know response options. Attitudes and practices were evaluated using a five‐point scale. Descriptive statistics were used to summarise the data collected. Logistic regression analysis was performed to identify the predictors of good food safety practices.

**Results:**

The mean knowledge score was 8.21 ± 2.13 out of 12. Good knowledge was observed in 39.8% of the participants, whereas 51.5% demonstrated a positive attitude towards food safety. However, only 34.5% of the respondents reported good food safety practices. Knowledge scores were positively correlated with practice scores (*ρ* = 0.41, *p* < 0.001), and attitude scores were positively correlated with practice scores (*ρ* = 0.36, *p* < 0.001). Higher knowledge scores (adjusted odds ratio [AOR]: 1.38; 95% confidence interval [CI]: 1.22–1.56; *p* < 0.001) and previous food safety training (AOR: 1.74; 95% CI: 1.18–2.56; *p* = 0.004) were significant predictors of good food safety practice.

**Conclusion:**

University students demonstrated moderate food safety knowledge and generally positive attitudes; however, hygienic food‐handling practices were less consistently followed. Strengthening food safety education and awareness programmes among university students may improve food safety behaviours and help reduce the risk of foodborne diseases.

## Graphical abstract

An online cross‐sectional survey of 412 university students in Rajasthan found moderate food safety knowledge and positive attitudes, but only one‐third demonstrated good food safety practices. Higher knowledge and food safety training predicted better practices, highlighting the need for practical, behaviour‐focused food safety education in universities.

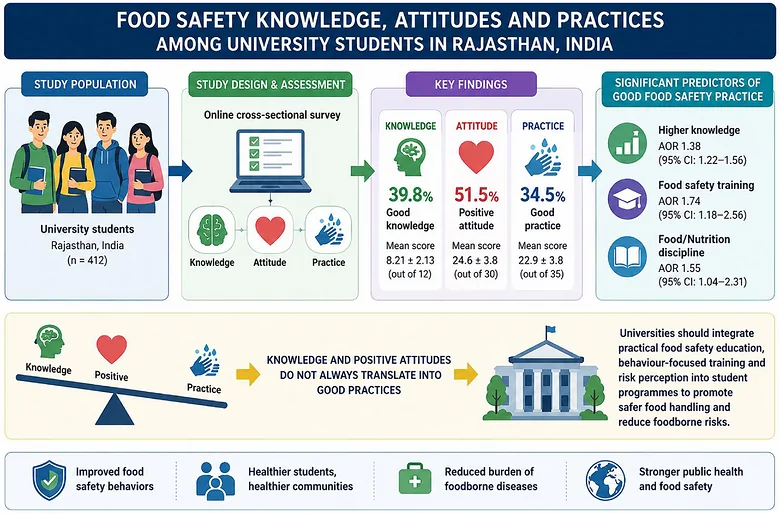

## 1 Introduction

Foodborne diseases remain a major global health issue, often related to poor food handling, low hygiene standards and a general lack of information on food safety [1]. According to recent estimates by the World Health Organization, unsafe food causes approximately 866 million illnesses and 1.5 million deaths annually worldwide, highlighting the substantial burden associated with foodborne diseases. In addition to their adverse health consequences, foodborne diseases impose substantial economic burdens through increased healthcare expenditures, productivity losses, reduced educational performance and disruptions to tourism and food trade. Young adults, especially university students, tend to prepare or eat food independently, which makes them vulnerable to food safety risks [2]. Improper storage, holding temperature and poor personal hygiene are major contributing factors for foodborne disease outbreaks. Thus, the most important step in reducing the risk of foodborne diseases and promoting overall healthier eating habits in this demographic is increasing the awareness of responsible food‐handling practices [3, 4, 5].

Although food safety research has traditionally concentrated on food handlers and vendors [6, 7, 8, 9], several studies have investigated food safety knowledge, attitudes and practices among university students in different countries [10, 11, 12]. However, consumer‐focused evidence from low‐ and middle‐income settings remains limited, and findings regarding the relationships between knowledge, attitudes and practices have been inconsistent. The results of these studies demonstrate diverse degrees of knowledge and significant discrepancies in the use of hygienic food‐handling practices [13]. Although a significant percentage of students demonstrate positive attitudes towards food safety, there are still apparent gaps in knowledge, especially in the areas of temperature control, cross‐contamination prevention and safe food storage [11, 14]. Although some studies have demonstrated that adequate food safety knowledge and positive attitudes are associated with improved hygienic food‐handling practices [15, 16], others have found weak or non‐significant relationships between these factors [17]. These inconsistencies suggest that the traditional Knowledge–Attitude–Practice (KAP) framework may not fully explain food safety behaviours. Although increased awareness is often assumed to promote safer practices, foodborne illnesses continue to occur because behavioural compliance is influenced by factors beyond knowledge alone, including motivation and commitments [3]. Accordingly, the recently proposed Knowledge–Attitude–Commitment–Practice (KACP) framework emphasizes the importance of motivation and commitment in promoting sustained behavioural change and improving compliance with hygienic food‐handling practices [18].

Despite the increasing interest in this area, limited evidence is available regarding food safety awareness among university students in many parts of India. Therefore, this study aimed to assess food safety knowledge, attitudes and practices among university students in Rajasthan, India. This study also aimed to identify factors associated with hygienic food‐handling practices among students to provide evidence that may support the development of targeted food safety education programmes.

## 2 Methods

### 2.1 Study Design, Study Participants and Study Duration

This cross‐sectional study was conducted among university students in Rajasthan, India to assess food safety knowledge, attitudes and practices. Data were collected over 3 months, from September to November 2025, using an English‐language questionnaire through online channels.

### 2.2 Inclusion and Exclusion Criteria

The study included students aged 18 years and above who were enrolled in universities in Rajasthan and were willing to participate in the study. Students who declined to provide consent or did not complete the questionnaires were excluded from the analysis.

### 2.3 Sample Size and Sampling Method

The sample size was calculated using the following formula for cross‐sectional studies:

$$n=Z^{2}P\left(1-P\right)/d^{2}$$

where *Z* represents the *Z*‐score at a 95% confidence level (1.96), *P* represents the estimated prevalence of adequate food safety knowledge (0.5 due to a lack of previous regional estimates), and *d* represents the margin of error (0.05). The minimum sample size calculated was 384. A large number of students were invited to fill out the questionnaire to account for potential non‐response. Finally, complete responses from 412 participants were included in the study.

Non‐probability convenience sampling was used to recruit participants. The survey link was shared via university communication channels, academic networks and student groups to promote voluntary participation.

### 2.4 Methods of Data Collection and Protocols Followed

Data were collected using a structured, self‐administered questionnaire consisting of four sections: sociodemographic characteristics, food safety knowledge, attitudes towards food safety and food safety practices. The questionnaire was designed following the ‘Five keys to safer food’ document produced by the World Health Organization and adapted from previously published food safety KAP instruments, with modifications to suit the Indian university context [19, 20, 21].

The questionnaire was reviewed by subject experts (*n* = 5) to ensure content validity, clarity and relevance. The instrument was assessed for clarity and feasibility through a pilot study with a small number of students (*n* = 30). It took approximately 8–10 min to complete the questionnaire. The items were tested by experts and a pilot study to ensure their relevance and understandability in the context of India, including the common practices of food handling, street food and the local food regulatory environment. Some minor changes were made on the basis of the feedback received from the pilot study, such as simplifying the language, clarifying the temperature‐related statements and sequencing the items in the questionnaire. Pilot study data were not analysed as part of the overall analysis.

For the final data collection, the participants were instructed to complete the questionnaire independently and honestly by selecting the responses that best reflected their knowledge, attitudes and routine practices without consulting external sources.

### 2.5 Scoring and Categorisation of KAP

The knowledge section consisted of 12 statements with response options of ‘true’, ‘false’ and ‘do not know’. The knowledge section comprised 12 items, with one point assigned to each correct response and zero points assigned to incorrect or *don't know* responses, yielding a total score ranging from 0 to 12. Knowledge scores were categorized as good (9–12 points), moderate (6–8 points) and poor (0–5 points). Attitudes were assessed using a five‐point Likert scale ranging from ‘strongly disagree’ (1 point) to ‘strongly agree’ (5 points), and food safety practices were evaluated using a five‐point frequency scale ranging from ‘never’ (1 point) to ‘always’ (5 points). Attitude and practice scores were derived from six items each, resulting in total scores ranging from 6 to 30. Attitude scores were classified as ‘positive’ (24–30 points), ‘neutral’ (18–23 points) and ‘negative’ (<18 points), whereas practice scores were categorized as ‘good’ (≥24 points), ‘moderate’ (18–23 points) and ‘poor’ (<18 points). These thresholds were adapted from scoring approaches used in previous KAP studies and were based on the percentage distributions of the maximum attainable scores to facilitate the interpretation and comparison of the findings.

This format was considered appropriate because it captures varying degrees of agreement and frequency while remaining simple, easy to understand and widely used in food safety KAP studies [11, 20, 21].

### 2.6 Statistical Analysis and Software Used

Data were exported to Microsoft Excel for cleaning and then analysed using the IBM Statistical Package for Social Sciences, version 25. Descriptive statistics, including frequencies, percentages, means and standard deviations, were used to summarise the participant characteristics and KAP scores. Independent‐samples *t*‐test was used to compare mean KAP scores between two‐category variables, whereas one‐way analysis of variance (ANOVA) was used for variables with more than two categories.

Spearman's rank correlation test was used to examine the relationships among KAP. Multivariate logistic regression analysis was conducted to explore the predictors of proper food safety practices. Variables that exhibited statistically significant relationships in the preliminary analysis and those that were theoretically relevant to food safety behaviours as reported in previous literature were included in the regression model. Accordingly, in the final regression model, the knowledge score, previous food safety training and field of study (Food/Nutrition vs. others) were included. Statistical significance was taken as *p* < 0.05 and adjusted odds ratio (AOR) along with 95% confidence interval (CI) was calculated.

## 3 Results

### 3.1 Characteristics of the Study Participants

A total of 412 university students participated in this study. The sociodemographic characteristics of the participants are presented in Table 1. Nearly half of the respondents (47.1%) were aged 21–23 years, whereas 29.1% were aged 18–20 years. There was a predominance of females in the study (56.8%), whereas males accounted for 41.3%. The majority of the participants were undergraduates (65.0%), whereas 30.1% were postgraduates, and 4.9% were pursuing PhDs. The disciplines most represented in the study were science (22.8%), engineering (19.9%), food and nutrition (17.5%), arts (16.0%) and commerce (15.5%). Regarding accommodation status, 35.4% of the participants lived in hostels, whereas 30.1% were living with their families. Only 26.2% of respondents reported having received formal food safety training, whereas the majority (73.8%) had not received any such training.

**TABLE 1:** Sociodemographic characteristics of participants (*n* = 412).

Variable	Category	*n*	%
Age group (years)	18–20	120	29.1
	21–23	194	47.1
	24–26	80	19.4
	≥27	18	4.4
Gender	Male	170	41.3
	Female	234	56.8
	Other	8	1.9
Level of study	Undergraduate	268	65.0
	Postgraduate	124	30.1
	PhD	20	4.9
Field of study	Food/Nutrition	72	17.5
	Science	94	22.8
	Commerce	64	15.5
	Arts	66	16.0
	Engineering	82	19.9
	Others	34	8.3
Residence type	Hostel	146	35.4
	Paying guest	74	18.0
	Rented accommodation	68	16.5
	With family	124	30.1
Food safety training	Yes	108	26.2
	No	304	73.8

### 3.2 Food Safety Knowledge, Attitudes and Practices

Table 2 presents the participants’ responses to the individual food safety knowledge statements. Overall, respondents demonstrated a high level of awareness regarding basic food hygiene practices, particularly the importance of handwashing with soap to reduce contamination (92.4%) and the need to store raw and cooked foods separately (82.0%). A substantial proportion also correctly identified that food poisoning is mainly caused by microorganisms (78.6%), that food allergies may cause severe reactions (76.2%) and that refrigeration slows bacterial growth (74.8%).

**TABLE 2:** Responses to food safety knowledge statements among university students (*n* = 412).

Knowledge statement	True (*n* [%])	False (*n* [%])	Don't know (*n* [%])
Food poisoning is mainly caused by microorganisms	324 (78.6)	38 (9.2)	50 (12.1)
Raw and cooked foods should be stored separately	338 (82.0)	26 (6.3)	48 (11.7)
Cooked food can be safely kept overnight at room temperature	72 (17.5)	284 (68.9)	56 (13.6)
Handwashing with soap reduces contamination	381 (92.4)	10 (2.4)	21 (5.1)
Refrigeration slows bacterial growth	308 (74.8)	44 (10.7)	60 (14.6)
Reheating leftovers reduces foodborne risk	287 (69.7)	61 (14.8)	64 (15.5)
Perishable foods should be stored ≤5°C	266 (64.6)	69 (16.7)	77 (18.7)
Same cutting board for raw and cooked foods	88 (21.4)	289 (70.1)	35 (8.5)
Thawing meat at room temperature increases risk	278 (67.5)	71 (17.2)	63 (15.3)
Food that looks normal is always safe	101 (24.5)	253 (61.4)	58 (14.1)
Food businesses require licensing in India	298 (72.3)	46 (11.2)	68 (16.5)
Food allergies may cause severe reactions	314 (76.2)	34 (8.3)	64 (15.5)

Moderate levels of knowledge were observed regarding food business licensing requirements in India (72.3%), the role of reheating leftovers in reducing foodborne risks (69.7%) and the increased risk associated with thawing meat at room temperature (67.5%). Knowledge was comparatively low for safe food storage temperatures, with only 64.6% correctly identifying that perishable foods should be stored at ≤5°C.

The lowest levels of knowledge were observed for statements related to cross‐contamination and food safety misconceptions. Only 21.4% correctly recognized that the same cutting board should not be used for both raw and cooked foods, whereas only 24.5% correctly disagreed with the statement that food appearing normal is always safe to consume.

Attitudes towards food safety were generally positive among the participants (Table 3). Most respondents agreed that food safety is important for maintaining health (90.5%). A large majority also supported the inclusion of food safety education for students (88.3%), compliance with food safety regulations by food businesses (86.4%) and the importance of checking expiry dates before food consumption (84.7%). Furthermore, 80.1% of participants agreed that street food hygiene is important, whereas 72.6% reported concern about experiencing food poisoning.

**TABLE 3:** Attitudes towards food safety among university students (*n* = 412).

Statement	Agree (*n* [%])	Neutral (*n* [%])	Disagree (*n* [%])
Food safety is important for maintaining health	373 (90.5)	29 (7.0)	10 (2.4)
Concern about getting food poisoning	299 (72.6)	76 (18.4)	37 (9.0)
Checking expiry dates is necessary	349 (84.7)	43 (10.4)	20 (4.9)
Students should receive food safety education	364 (88.3)	34 (8.2)	14 (3.5)
Food businesses must follow safety regulations	356 (86.4)	38 (9.2)	18 (4.4)
Street food hygiene is important	330 (80.1)	52 (12.6)	30 (7.3)

The participants’ self‐reported food safety practices are shown in Table 4. Washing of fruits and vegetables before consumption (71.3% always) and washing of hands before eating/food preparation (68.4% always) had high levels of compliance. Majority of students also reported that they always check the expiry date before buying food (65.0%) and always avoid the food vendors selling food on the streets which was visibly unhygienic (54.2%). Food storage practices, however, were somewhat lower with just 48.6% of the participants saying that they always refrigerated leftovers within 2 h. Likewise, 52.1% said they always reheat leftovers before eating.

**TABLE 4:** Self‐reported food safety practices among students (*n* = 412).

Practice	Always (%)	Often (%)	Sometimes (%)	Rarely (%)	Never (%)
Wash hands before eating/cooking	68.4	18.9	7.3	3.1	2.3
Refrigerate leftovers within 2 h	48.6	23.5	14.1	8.2	5.6
Reheat leftovers before eating	52.1	21.4	13.8	7.7	5.0
Wash fruits and vegetables	71.3	16.7	6.9	3.1	2.0
Check expiry dates before purchase	65.0	20.9	8.7	3.8	1.6
Avoid unhygienic street vendors	54.2	19.4	13.3	8.5	4.6

Table 5 presents the overall food safety knowledge, attitude and practice levels of the participants. The mean knowledge, attitude and practice scores were 8.21 ± 2.13, 24.6 ± 3.8 and 22.9 ± 4.7, respectively. Although over half of the respondents (51.5%) demonstrated positive attitudes towards food safety, only 39.8% exhibited good knowledge, and 34.5% reported good food safety practices.

**TABLE 5:** Overall food safety knowledge, attitude and practice levels (*n* = 412).

Variable	Category	Score range	*n*	%	Mean ± SD
Knowledge	Good	9–12	164	39.8	8.21 ± 2.13
	Moderate	6–8	158	38.3	
	Poor	0–5	90	21.9	
Attitude	Positive	24–30	212	51.5	24.6 ± 3.8
	Neutral	18–23	136	33.0	
	Negative	<18	64	15.5	
Practice	Good	≥24	142	34.5	22.9 ± 4.7
	Moderate	18–23	170	41.3	
	Poor	<18	100	24.2	

### 3.3 Association Between Knowledge, Attitudes and Practices

Table 6 presents the correlation and regression analyses of factors associated with food safety practices. Knowledge scores were positively correlated with practice scores (*ρ* = 0.41, *p* < 0.001), whereas attitude scores also showed a significant positive correlation with food safety practices (*ρ* = 0.36, *p* < 0.001). In the multivariable logistic regression analysis, higher knowledge scores (AOR = 1.38, 95% CI: 1.22–1.56, *p* < 0.001), previous food safety training (AOR = 1.74, 95% CI: 1.18–2.56, *p* = 0.004) and enrolment in a Food/Nutrition‐related discipline (AOR = 1.55, 95% CI: 1.02–2.34, *p* = 0.039) were significant predictors of good food safety practices among university students.

**TABLE 6:** Association and regression analysis for predictors of good food safety practice.

Variable	Spearman *ρ*/AOR	95% CI	*p* value
Knowledge–Practice correlation	0.41	—	<0.001
Attitude–Practice correlation	0.36	—	<0.001
Knowledge score (regression)	1.38	1.22–1.56	<0.001
Food safety training	1.74	1.18–2.56	0.004
Food/Nutrition discipline	1.55	1.02–2.34	0.039

Abbreviations: AOR, adjusted odds ratio; CI, confidence interval.

### 3.4 Sociodemographic Characteristics and KAP Scores

Food safety knowledge differed significantly according to field of study, with the highest mean knowledge score observed among students from Food/Nutrition disciplines (9.05 ± 1.76; *p* = 0.004). Students who had received food safety training had significantly higher knowledge scores than those who had not received training (8.82 ± 1.87 vs. 8.00 ± 2.18; *p* < 0.001) and higher practice scores (24.05 ± 4.31 vs. 22.46 ± 4.77; *p* = 0.002). Attitude scores also differed significantly according to residence type (*p* = 0.020), with the highest mean attitude score observed among students living in rented accommodation (25.32 ± 3.54). No statistically significant differences in KAP scores were observed across the other sociodemographic characteristics (Table 7).

**TABLE 7:** Association of sociodemographic characteristics with food safety knowledge, attitude and practice scores.

Variable	Category	Knowledge mean ± SD	*p* value	Attitude mean ± SD	*p* value	Practice mean ± SD	*p* value
Age group (years)	18–20	8.02 ± 2.17		24.09 ± 3.78		22.34 ± 4.66	
	21–23	8.18 ± 2.11	0.389	24.42 ± 3.73	0.304	22.77 ± 4.61	0.226
	24–26	8.45 ± 2.03		24.93 ± 3.68		23.51 ± 4.53	
	≥27	8.70 ± 1.97		25.41 ± 3.60		24.03 ± 4.38	
Gender	Male	8.11 ± 2.14		24.40 ± 3.75		22.69 ± 4.68	
	Female	8.34 ± 2.05	0.523	24.79 ± 3.70	0.559	23.11 ± 4.57	0.648
	Other	8.48 ± 2.01		25.01 ± 3.79		23.32 ± 4.62	
Level of study	Undergraduate	8.13 ± 2.12		24.42 ± 3.76		22.74 ± 4.65	
	Postgraduate	8.36 ± 2.05	0.381	24.75 ± 3.71	0.492	23.17 ± 4.53	0.487
	PhD	8.66 ± 1.95		25.27 ± 3.56		23.76 ± 4.36	
Field of study	Food/Nutrition	9.05 ± 1.76		25.34 ± 3.40		24.22 ± 4.14	
	Science	8.59 ± 1.99		24.80 ± 3.59		23.33 ± 4.45	
	Commerce	8.03 ± 2.15	0.004*	24.25 ± 3.83	0.321	22.52 ± 4.69	0.095
	Arts	7.84 ± 2.18		24.03 ± 3.89		22.19 ± 4.73	
	Engineering	8.09 ± 2.10		24.45 ± 3.72		22.74 ± 4.58	
	Others	7.98 ± 2.16		24.17 ± 3.84		22.39 ± 4.66	
Residence type	Hostel	8.04 ± 2.13		23.86 ± 3.67		22.58 ± 4.66	
	Paying guest	8.37 ± 2.02	0.401	25.11 ± 3.56	0.020*	23.23 ± 4.49	0.506
	Rented accommodation	8.52 ± 1.98		25.32 ± 3.54		23.53 ± 4.46	
	With family	8.18 ± 2.10		24.45 ± 3.79		22.86 ± 4.69	
Food safety training	Yes	8.82 ± 1.87		24.98 ± 3.50		24.05 ± 4.31	
	No	8.00 ± 2.18	<0.001*	24.46 ± 3.88	0.221	22.46 ± 4.77	0.002*

* indicates statistical significance at p < 0.05.

## 4 Discussion

This study assessed food safety knowledge, attitudes and practices among university students in Rajasthan, India. The findings indicate that although participants generally demonstrated moderate food safety knowledge and positive attitudes towards food safety, good food safety practices were reported by only one‐third of respondents. Furthermore, higher knowledge scores, previous food safety training and enrolment in Food/Nutrition‐related disciplines were identified as significant predictors of good food safety practices.

The moderate level of food safety knowledge observed in this study is consistent with findings reported among university students in Bangladesh, Pakistan and other South Asian countries [22, 23, 24]. Although respondents demonstrated good awareness of basic food hygiene practices, such as handwashing and the separation of raw and cooked foods, important knowledge gaps were identified regarding safe storage temperatures, cross‐contamination and the identification of potentially unsafe foods. Similar deficiencies have been reported by Sultana et al. [21], who found that young adults often possess basic food safety knowledge but have limited understanding of specific food handling and storage requirements. This may be because university students are more frequently exposed to general health and nutrition information than to structured food safety education. Consequently, practical aspects, such as temperature control, cross‐contamination prevention and safe food storage, may receive less attention than basic hygiene practices.

University students generally exhibited positive attitudes towards food safety, with most recognising the importance of food safety for health, food safety education and compliance with food safety regulations. These findings are consistent with previous studies reporting favourable food safety attitudes among students and young adults [25, 26]. Positive attitudes are important because they may facilitate the adoption of hygienic food‐handling behaviours when supported by adequate knowledge, training and opportunities for skill development [27, 28].

Despite the generally positive attitudes observed, food safety practices were less consistently followed. Although most participants reported regularly washing hands, cleaning fruits and vegetables and checking expiry dates, practices related to safe food storage and handling of leftovers were less frequently reported. This discrepancy between knowledge, attitudes and behaviour has been widely documented in food safety research. Several studies have shown that individuals may possess basic food safety knowledge but fail to apply it consistently in daily practice because of factors, such as convenience, lack of training and limited perception of foodborne disease risk [29]. Among university students, busy academic schedules, frequent consumption of food prepared outside the home and limited involvement in routine food preparation may further hinder the translation of knowledge into consistent food safety practices.

The positive correlations found between knowledge, attitudes and food safety practices are significant, which supports the relationships suggested in the traditional KAP model. The moderate strength of these associations, however, indicates that simply knowing is not enough to ensure behavioural compliance. Although participants generally had a moderate level of knowledge and positive attitudes to food safety, only one‐third reported good food safety practices. This result is in line with the increasing body of evidence that behaviours are determined by factors other than knowledge and attitudes, such as motivation, commitment, risk perception and habits [3]. The traditional KAP model has recently been expanded to include commitment and motivation, as part of KACP models, which has further emphasised the role of commitment and motivation in enabling knowledge to lead to sustained behaviour change or improved compliance with hygienic food‐handling practices [18]. Hence, food safety interventions should go beyond knowledge improvement to reinforce individual food safety commitment and the intention to consistently adopt safe food‐handling practices.

Various psychological and behavioural variables, such as risk perception, affect compliance with recommended behaviours for food safety. People might have the overconfidence bias and think that with past experience they know how to work safely with food even if they are not using recommended practices [30]. Some people might show optimistic bias, believing that food poisoning will not happen to them. These perceptions can also lower the level of motivation to engage in hygienic food‐handling behaviours and even among those who have good knowledge and positive attitudes [31, 32, 33]. The results indicate that in the future the interventions must also focus on behavioural determinants and risk perception as well as increasing knowledge.

An important finding of the present study was the association between food safety training and improved food‐handling practices [34]. Students who received formal food safety training were more likely to demonstrate safe food practices. Similar associations have been reported previously, highlighting the important role of structured education and training programmes in improving food safety awareness and behavioural outcomes [34, 35, 36]. The association observed among students enrolled in Food/Nutrition‐related disciplines further supports the influence of educational exposure on food safety behaviours.

The present study contributes to the existing literature by providing evidence on food safety knowledge, attitudes and practices among university students in an Indian setting, where such data remain limited. The findings further support growing evidence that the relationship between knowledge and behaviour may not be linear, suggesting that improving awareness alone may be insufficient to ensure consistent hygienic food‐handling practices. From a practical perspective, the results highlight the need for behavioural and skill‐based food safety interventions, including the integration of food safety education into university curricula, orientation programmes and health promotion initiatives [37]. Such interventions may help strengthen risk perception, encourage positive behavioural change and ultimately contribute to reducing the burden of foodborne diseases. Future multicentre studies using probability‐based sampling across different regions of India are further warranted to validate and extend the present findings.

### 4.1 Strengths and Limitations

A key strength of present study is its consumer's perspective to food safety issues, as there are very few studies in the field of food safety that have included a consumer focus. By examining KAP among university students, the study provides evidence on food safety behaviours among young adult consumers in a low‐ and middle‐income setting. To the best of our knowledge, this study is the first study that investigated university student's food safety–related knowledge, attitudes and practices in Rajasthan, India.

Nevertheless, this study had several limitations also. The cross‐sectional design precluded the ability to make causal inference between knowledge, attitudes and practices. Non‐probability sampling (convenience sampling) might also affect the representativeness of the results and risk selection bias. As open dissemination was used to distribute the survey, an exact response rate was not calculated and may have resulted in selection bias, thus reducing the representativeness of the survey sample population. Thus, the results should be treated with caution and may not be representative of all university students at Rajasthan or in other parts of India. Moreover, factors that could have confounded the findings, such as socioeconomic status, prior exposure to food safety information and living arrangements, were not fully examined.

## 5 Conclusion

The findings of the present study suggest that the knowledge regarding food safety among university students in Rajasthan was moderate, and most of the students had a positive attitude towards food safety, but only one‐third reported good food safety practices. There was a positive correlation between food‐handling practices and higher knowledge levels, as well as experience in food safety training, which is an important finding. Therefore, the results indicate that knowledge might not be enough to assure the same hygienic food‐handling behaviours and that aspects of the behaviours, such as risk perception and individual commitment, might affect compliance with recommended food safe‐handling practices. Food safety education and the development of food safety skills should be incorporated into the curriculum and student health promotion programmes at universities, especially in the areas of safe food storage, prevention of cross‐contamination and raising awareness of foodborne diseases. The use of such interventions could encourage safer food‐handling practices and help lower the foodborne disease burden in young adults.

## Author Contributions

**Naresh Kumar Sharma**: data curation, formal analysis, writing – original draft. **Naveen Kumar**: conceptualisation, methodology, data curation, formal analysis, writing – original draft, supervision. **Ravinder Kaushik**: writing – review and editing. **Vijay Pal Singh**: writing – review and editing. **Sanjeeb Kumar Mishra**: conceptualisation, supervision, writing – review and editing. All authors reviewed and approved the final manuscript.

## Funding

The authors have nothing to report.

## Ethics Statement

The study was approved by the Institutional Human Ethics Committee (K.R. Mangalam Ethics Committee for Human Participants), with reference number KRMU/RDC/2026‐27/0179. Data collection was performed following the principles of the Declaration of Helsinki (2013).

## Consent

Participation was voluntary, and informed digital consent was obtained from all participants before they completed the questionnaire. The purpose of the study was explained in writing to all participants while circulating the questionnaire. Personal identification details were not collected, and the confidentiality of the responses was maintained throughout the study.

## Conflicts of Interest

The authors declare no conflicts of interest.

## Data Availability

Most of the data generated in this study are presented in Section 3 through tables. Additional data can be obtained from the corresponding authors upon reasonable request.
